# The CAP study, evaluation of integrated universal and selective prevention strategies for youth alcohol misuse: study protocol of a cluster randomized controlled trial

**DOI:** 10.1186/1471-244X-12-118

**Published:** 2012-08-20

**Authors:** Nicola C Newton, Maree Teesson, Emma L Barrett, Tim Slade, Patricia J Conrod

**Affiliations:** 1 National Drug and Alcohol Research Centre, University of New South Wales, 22-32 King Street, Randwick, NSW, 2052, Australia; 2 Department of Psychiatry, Université de Montréal, Montreal, Canada

**Keywords:** Prevention, School, Internet, Personality, Selective, Universal, Randomized controlled trial

## Abstract

**Background:**

Alcohol misuse amongst young people is a serious concern. The need for effective prevention is clear, yet there appear to be few evidenced-based programs that prevent alcohol misuse and none that target both high and low-risk youth. The CAP study addresses this gap by evaluating the efficacy of an integrated approach to alcohol misuse prevention, which combines the effective universal internet-based *Climate Schools* program with the effective selective personality-targeted *Preventure* program. This article describes the development and protocol of the CAP study which aims to prevent alcohol misuse and related harms in Australian adolescents.

**Methods/Design:**

A cluster randomized controlled trial (RCT) is being conducted with Year 8 students aged 13 to 14-years-old from 27 secondary schools in New South Wales and Victoria, Australia. Blocked randomisation was used to assign schools to one of four groups; *Climate Schools* only, *Preventure* only, CAP (*Climate Schools and Preventure*), or Control (alcohol, drug and health education as usual). The primary outcomes of the trial will be the uptake and harmful use of alcohol and alcohol related harms. Secondary outcomes will include alcohol and cannabis related knowledge, cannabis related harms, intentions to use, and mental health symptomatology. All participants will complete assessments on five occasions; baseline; immediately post intervention, and at 12, 24 and 36 months post baseline.

**Discussion:**

This study protocol presents the design and current implementation of a cluster RCT to evaluate the efficacy of the CAP study; an integrated universal and selective approach to prevent alcohol use and related harms among adolescents. Compared to students who receive the stand-alone universal *Climate Schools* program or alcohol and drug education as usual (Controls), we expect the students who receive the CAP intervention to have significantly less uptake of alcohol use, a reduction in average alcohol consumption, a reduction in frequency of binge drinking, and a reduction in alcohol related harms.

**Trial registration:**

This trial is registered with the Australian and New Zealand Clinical Trials registry, ACTRN12612000026820.

## Background

Alcohol misuse amongst young people is common and the burden of disease, social costs, and disability associated with this use is considerable [1-4]. The peak of this disability occurs in those aged 15–24 years and corresponds with the typical age of initiation to alcohol and other drug use [5]. The high prevalence of use amongst adolescents is of particular concern given that early initiation to substance use is a risk factor for the development of substance use disorders, co‐morbid mental health problems, juvenile offending, impaired educational performance and early school drop‐out, all of which negatively impact on both current functioning and future life options [6-8].

To reduce the occurrence and cost of such problems, prevention is essential and needs to be initiated early before harmful patterns of alcohol and other drug use are established and begin to cause disability [9,10]. Although an array of school-based prevention programs exist [11-16], the majority show minimal effects in reducing alcohol use and related harms [17-20], and some have even reported iatrogenic effects [21,22]. The most common factor which impedes on effectiveness is implementation failure [23-25]. Given that school‐based prevention is the primary means by which alcohol and other drug education is delivered, it is essential to focus on increasing program efficacy.

There are two common approaches to school-based drug education; ‘universal’ and ‘selective’ [26]. The selective approach involves delivering programs which target specific populations, such as individuals at greatest risk for developing substance use problems. On the other hand, the universal approach aims to deliver interventions to all students regardless of their level of risk for drug use, and focus largely on teaching normative education and drug resistance skills [27]. Ideally, preventive interventions should aim to delay onset in both adolescents with low-risk profiles who may be influenced to take up alcohol and other drugs due to peer influence and social conformity, and adolescents with high-risk profiles whose underlying vulnerability to psychopathology can lead to substance misuse. Yet, there appear to be no models of well implemented programs that do this. The current cluster randomised controlled trial (RCT) addresses this gap by developing and evaluating an integrated approach to preventing alcohol misuse and related harms in adolescents by combining the efficacious ‘universal’ *Climate Schools* and ‘selective’ *Preventure* programs.

### The universal ‘Climate Schools’ program

The universal *Climate Schools* program aims to reduce the use of the most commonly used licit and illicit drugs in most developed countries: alcohol and cannabis [2,4]. The *Climate Schools* program is based on the effective harm-minimisation approach to prevention [28-32] and uses cartoon storylines to engage and maintain student interest and involvement over time. The program is facilitated by the internet which guarantees complete and consistent delivery whilst ensuring high implementation fidelity. The program is designed to fit within the school health curriculum and be implemented to students 13–14 years old before significant exposure to alcohol and other drug use occurs. The *Climate Schools* program consists of twelve 40-minute lessons; the first six lessons focus specifically on alcohol and are delivered approximately six month prior to the remaining six lessons which focus on both alcohol and cannabis.

The first part of each lesson is completed individually over the internet where students navigate through cartoon storylines which impart information about the short- and long-term effects of alcohol and cannabis, normative alcohol and cannabis use, drug refusal and harm-minimisation skills, and tips on staying safe and first aid. Students are provided with confidential login details to access the *Climate Schools* website. The second part of each lesson is a group or class activity delivered by the teacher which reinforces the information in the cartoons and allows interactive communication between students. Teachers are provided with a manual containing the activities, implementation guidelines, links to the education syllabus and teacher and student summaries for each lesson.

The efficacy of the *Climate Schools* program has been established using a cluster RCT in 10 schools in Sydney, Australia (n = 764) [31-33]. Results of the trial demonstrated that compared to the control group, students in the intervention group showed significant improvements in alcohol and cannabis knowledge at the end of the course and at six and twelve months following the intervention. In terms of behaviour change, the intervention group showed a reduction in frequency of cannabis use at the six-month follow-up, a reduction in average weekly alcohol consumption at the six and twelve month follow-ups, and a reduction in frequency of drinking to excess twelve months following the intervention. In addition, program evaluation showed that students and teachers rated the program as an acceptable and enjoyable means of delivering drug education in schools. Specifically, 100% of teachers who implemented this program in their classroom rated it as superior to other drug prevention programs, and over 90% of students reported information delivered in this format was easy to learn and would like more school subjects to be taught through this method.

Despite these positive results, the effectiveness of the *Climate Schools* program is somewhat limited. Firstly, the *Climate Schools* program is intended only to reduce the use of alcohol and cannabis and not other drugs. As the prevalence of illicit drug use other than cannabis is relatively low amongst adolescents, it has been suggested that such drugs may be better addressed using selective rather than universal prevention programs [34]. Secondly, although the *Climate Schools* program had significant effects on reducing alcohol and cannabis use, the effect sizes were modest (<0.38) [35], as is expected with universal programs [12,16]. In addition, analyses of the efficacy of *Climate Schools* in high-risk students only (i.e., youth already using substances or youth with substance using peers), found the effects to be smaller than those high-risk students experience as a result of participating in ‘selective’ interventions [36,37]. This could be attributed to the fact that most universal preventive interventions address substance use through a social influence perspective and do not take into account the many other risk factors involved in developing substance use disorders such as underlying vulnerabilities due to individual and genetic factors [38]. This suggests that high-risk students may benefit from additional ‘selective’ prevention which is specifically tailored to their needs and risk factors. Selective programs offer the benefit of being able to focus on the role of other risk factors for substance use such as psychopathology and personality. Such programs have often been overlooked due to their practical limitations as not only is it difficult to initially identify those individuals at greatest risk, but finding suitable, cost-effective ways to screen and deliver interventions can also be challenging [26]. The selective personality-targeted *Preventure* program overcomes these obstacles.

### The selective ‘Preventure’ program

The school-based *Preventure* program is a brief manualised personality-targeted substance use preventive intervention for high-risk adolescents aged 13–15 years. *Preventure* is the first and only selective school-based program that has been shown to curb excessive alcohol and illicit drug use in Canada and the United Kingdom (UK) [37,39-41]. Unlike universal programs delivered to a whole population, this selective personality-targeted approach addresses four personality risk-factors for early-onset substance misuse and other risky behaviours: Sensation Seeking, Impulsivity, Anxiety Sensitivity and Negative Thinking [42]. The *Preventure* program is also consistent with new models which conceptualise substance use as being driven by personality traits such as impulsivity and disinhibition [43].

The *Preventure* program involves two 90-minute group sessions, specific to the four personality types, which are carried out by a trained facilitator and co-facilitator. The interventions are conducted using manuals which incorporate psycho-educational and cognitive behavioural components, and include real life scenarios shared by high-risk youth in specifically-organised focus groups. In the first session, participants are guided in a goal setting exercise, designed to enhance motivation to change behaviour. Psycho-educational strategies are used to teach participants about their target personality trait and associated problematic coping behaviours like avoidance, interpersonal dependence, aggression, risky behaviours and substance misuse. They are then introduced to the cognitive behavioural model and guided in breaking down personal experience according to the physical, cognitive and behavioural components of an emotional response. A novel component to this intervention approach is the fact that all exercises discuss thoughts, emotions and behaviours in a personality-specific way, e.g. identifying situational triggers and cognitive distortions related to Sensation Seeking specifically. In the second session, participants are encouraged to identify and challenge personality-specific cognitive distortions that lead to problematic behaviours.

The efficacy of the *Preventure* program has been demonstrated in a number of RCTs in Canada and the UK [36,37,40,41,44]. Results from these trials revealed that *Preventure* successfully stemmed the growth in drinking and binge drinking in high-risk youth at six- and twelve-months following the intervention [37], and more recent analysis has revealed the onset and escalation of drug misuse was prevented over a two-year period [41]. In addition, *Preventure* has been shown to reduce emotional and behavioural problems specific to each of the personality profiles [45]. This is of particular importance given that comorbidity between substance use disorders and ill mental health is substantial and leads to worse outcomes [8]. Finally, a recent effectiveness cluster RCT showed that a standardised training model which trained teachers to deliver the program in schools resulted in treatment effects that are comparable to those reported for the previous trial involving more controlled treatment delivery conditions [36].

## Objectives of the CAP study

The primary objective of the current study is to integrate the effective universal *Climate Schools* and selective *Preventure* programs into a comprehensive model to prevent alcohol misuse and related harms in adolescents. This model will be known as the ‘CAP *(Climate and Preventure)* intervention’ and will result in a sequential approach to drug prevention which overcomes traditional implementation obstacles to school-based prevention. Delivering prevention using the proposed comprehensive approach offers a way of preventing substance misuse at a whole population level and has the potential to maximize outcomes for both high- and low-risk youth. To our knowledge, in Australia there is currently no evidence of effective selective prevention and internationally there is no evidence of an integrated universal and selective approach to substance use prevention.

The primary aims of the study are to evaluate the efficacy of the integrated CAP intervention in comparison to stand-alone ‘universal’ prevention and treatment as usual (TAU), in reducing the uptake and harmful use of alcohol, and reducing alcohol related harms. In addition, for high-risk students only, we aim to evaluate the efficacy of the selective personality-targeted *Preventure* program in comparison to stand-alone ‘universal’ prevention and TAU, in reducing the uptake and harmful use of alcohol, and reducing alcohol related harms.

Secondary outcomes of the study will be examined and will include effects of the programs on increasing alcohol and cannabis related knowledge, reducing cannabis related harms, reducing intentions to use, and reducing mental health symptomatology.

## Methods/Design

In 2010, we were awarded a five year National Health and Medical Research Council of Australia (NHMRC) project grant to conduct the CAP study. Ethics approval was obtained by the University of New South Wales Human Ethics committee (HREC 11274), the Sydney Catholic Education Office (Ref: 772) and the NSW Department of Education and Training (SERAP 2011201).

### Modification of Preventure

In 2011 the Preventure interventions and student manuals which were originally developed for Canada and the UK were modified for use in Australia using feedback obtained from focus groups with young Australians. Eight focus groups were conducted in three Sydney high schools covering the four personality groups; Sensation Seeking, Impulsivity, Anxiety Sensitivity and Negative Thinking. In the focus groups, students were first asked to share their own experiences regarding alcohol and drugs and what would lead them to use alcohol and other drugs in the future. They were also asked to provide scenarios where teenagers are likely to drink alcohol and asked if their personality type was linked to reasons people drink alcohol. Following this, the student manuals were focus tested. The student manuals consist of text, exercises, and real-life experiences and scenarios that were originally generated by organized focus groups of high-risk personality adolescents. Students were asked to provide feedback on the content, the illustrations, and the scenarios in the manuals specific to their personality group. Using this feedback and scenarios provided the content and illustrations in the student manuals were modified for the cultural and school context of Australia.

### Study design

**Figure 1:**
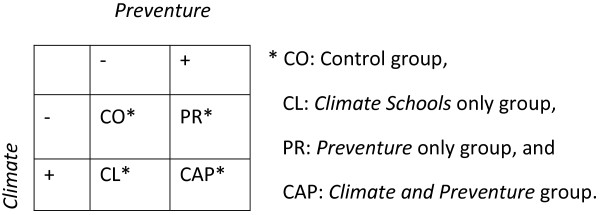
Experimental design of the CAP study.

Following the modification of *Preventure,* we seek to establish the efficacy of the integrated CAP intervention. To do this we are currently conducting a cluster RCT in Australian schools. Cluster randomisation was employed to avoid contamination of the control group with the intervention group through student communication. Participating schools have been randomly allocated to one of four groups; (1) the ‘Control’ condition (CO), (2) the ‘*Climate Schools* only’ condition (CL), (3) the ‘*Preventure* only’ condition (PR), or (4) the ‘*Climate and Preventure*’ condition (CAP). See Figure 1 for a graphical display of the experimental design.

### Sample size calculations

To account for cluster randomisation, sample size calculations were based on recent methods developed by Heo & Leon [46] to detect intervention by time interactions in longitudinal cluster RCTs. This trial is powered to detect differences between groups in the overall sample as well as in the high-risk students only. To allow for comparisons between the high-risk students and the overall sample, 600 high risk students from 20 schools were required (i.e. 30 high-risk students per school and 5 schools per intervention group). This achieves 80% power to detect a standardized between-group mean difference of 0.3 (p = 0.05) in outcomes at the end of the trial with 5 measurement occasions. An effect size of 0.3 is comparable to previous trials of drug prevention programs [12,16], and is the expected difference between the CAP and CL groups based on analyses of our previous research.

To account for dropouts during the trial which we expect to be approximately 10% [31,41], our initial aim was to recruit 80 students per school, 40% (n = 32) of whom were expected to be high-risk based on previous research by Dr. Conrod [37,39], and 6 schools per group giving us a total of 1920 students from 24 schools at baseline to test the effect of the intervention in the overall group.

### Recruitment of schools and randomisation

**Figure 2:**
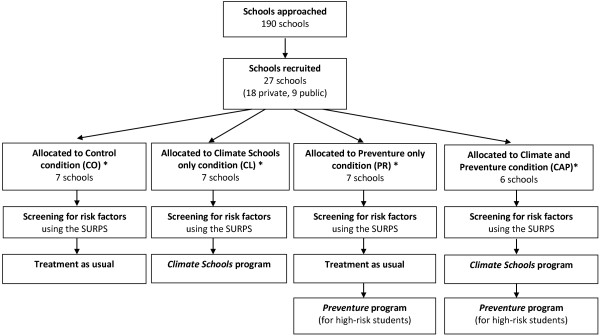
Recruitment and allocation of schools and students to the CAP study intervention groups.

The recruitment, inclusion, and randomization of the participants (schools and students) commenced in September 2011. A total of 190 schools were selected randomly from a list of all public and private secondary schools in New South Wales and Victoria. A letter outlining the aims of the research trial was sent to school principals. This letter provided information on what was required of their school if they agreed to participate, including time frames and information on the randomisation procedure. A total of 27 schools agreed to participate in the research. The main reasons for schools not participating were lack of time and no interest in participating in research in general. Following school consent, blocked randomisation occurred using the online program http://www.randomization.com, and schools were allocated to one of the four intervention groups shown in Figure 2. Information and consent forms were sent home to parents/guardians of all Year 8 students (ages 13–14 years) at participating schools, and only students who received parental consent (passive for private schools and active for public schools) and gave active consent themselves were eligible to be involved in the study.

### Interventions

Following the baseline assessment and the SURPS screening, students in the intervention groups are currently receiving; TAU, the *Climate Schools* program, the *Preventure* program, or the CAP intervention during Year 8.

The *Climate Schools* program consists of twelve 40-minute lessons aimed at reducing the use of alcohol and cannabis and related harms. The first six lessons focus specifically on alcohol and are delivered approximately six month prior to the remaining six lessons which focus on alcohol and cannabis. Students create unique confidential login details to access the CAP study website ( http://www.capstudy.org.au) where they complete the first part of each lesson in the form of a cartoon storyline which imparts information on alcohol and cannabis. The second part of each lesson is a group or class activity delivered by the teacher which reinforces the information in the cartoons and allows interactive communication between students. Teachers are provided with access to an online or hard copy of the teacher’s manual which contains activities, implementation guidelines, links to the syllabus and summaries for each lesson.

The *Preventure* intervention involves two 90 minute group sessions, carried out at the participant’s schools and delivered one week apart. The interventions are specific to each of the four personality profiles (SS, NT, AS and IMP) and only students who score ‘high-risk’ on any one of the four personality subscales on the SURPS are invited to receive the *Preventure* interventions. The interventions are provided by a qualified facilitator and a co-facilitator who were trained according to the training protocol described in O’Leary Barrett et al. [36]. The facilitators (registered clinical psychologists) and co-facilitators (minimum training, Bachelor of Psychology Honours degree) received three days training from Dr Conrod, who developed the original interventions. In addition, the facilitators were supervised by Dr Conrod in the delivery of the full intervention at two pilot schools with students in each personality high-risk group.

In the first *Preventure* group session, psycho-educational strategies are used to educate students about the target personality variable (NT, AS, IMP, or SS) and the associated problematic coping behaviours, such as interpersonal dependence, aggression, risky behaviours, and substance misuse. Students were motivated to explore their personality and ways of coping with their personality through a goal-setting exercise. Thereafter, they were introduced to the cognitive behavioural model by analysing a personal experience according to the physical, cognitive, and behavioural responses. In the second session, participants are encouraged to identify and challenge personality-specific cognitive thoughts that lead to problematic behaviours. For example, the impulsivity intervention focused on not thinking things through and aggressive thinking, and the sensation-seeking intervention focused on challenging cognitive thoughts associated with reward seeking and boredom susceptibility.

Students randomised to the *Control* group receive their usual health education classes (including lessons on drugs and alcohol) over the year. Control schools have been asked to record what drug education, if any, they deliver during the year including how many lessons and the format of the lessons. Control schools will be offered the use of the *CAP* intervention following completion of the study. See Figure 2 for intervention breakdown by group.

### Assessment occasions

Regardless of the condition to which schools were assigned, all students are assessed via a self-report questionnaire at baseline, immediately-post intervention (6 month follow up), and 12, 24 and 36 months after baseline. Students from 26 of the 27 schools have opted to complete assessments online via the CAP study website http://www.capstudy.org.au. Each student has a unique username and password to login to the website and all survey data obtained is strictly confidential. The remaining school opted to complete pen and paper surveys due to limited availability of computer resources. Table 1 displays the anticipated CAP study assessment times.

**Table 1:** Anticipated CAP study assessment times

	**Baseline Survey and SURPS**	***Climate Schools *program**	***Preventure *program (high-risk students)**	**6 month F/U Survey**	**12 month F/U Survey**	**24 month F/U Survey**	**36 month F/U Survey**
**Time**	**Feb-May 2012**	**Feb-Sept 2012**	**Feb-Sept 2012**	**Sept-Oct 2012**	**Feb-May 2013**	**Feb-May 2014**	**Feb-May 2015**
**Grade**	**Year 8**	**Year 8**	**Year 8**	**Year 8**	**Year 9**	**Year 10**	**Year 11**
CO*	✓			✓	✓	✓	✓
CL*	✓	✓		✓	✓	✓	✓
PR*	✓		✓	✓	✓	✓	✓
CAP*	✓	✓	✓	✓	✓	✓	✓

*CO: Control group, CL: *Climate* only group, PR: *Preventure* only group, and CAP: *Climate and Preventure* group.

## Measures

Demographic data including gender, age, academic performance, and truancy rates are obtained to determine baseline equivalence of the groups.

### Screening for high-risk students

During the baseline assessment, all students are screened for levels of personality risk to substance use using the Substance Use Risk Profile Scale (SURPS) [42]. The SURPS is a 23-item questionnaire assessing variation in personality risk for substance abuse/dependence along 4 dimensions: Sensation Seeking, Impulsivity, Anxiety Sensitivity and Negative Thinking. Students who score more than one SD above the school mean on any of the four personality risk subscales are categorized into these sub-groups. If a student scores a high score on more than one subscale, they are assigned to the group in which they show the most statistical deviance according to z-scores. Approximately 40% of students fall into one of these four sub groups. The SURPS has good concurrent, predictive and incremental validity with regards to differentiating individuals prone to reinforcement-specific patterns of substance use [37,39].

### Alcohol and other drug use

Alcohol use is assessed using a questionnaire adapted from the SHAHRP ‘Patterns of Alcohol’ index [47]. This measures frequency and quantity of consumption in standard drinks, and frequency of drinking to excess defined as having more than four standard drinks on a single occasion. Other drug use is measured based on the questions from the NDSHS 2010 [48]. This allows for comparison between use in the current sample and a large scale representative group of Australians.

### Alcohol and cannabis related harms

Alcohol related harms (in the past 6 months) are assessed using an abbreviated version of the Rutgers Alcohol Problem Index (RAPI) [49], based on the most frequently endorsed problems by adolescents age 14–16 years from Preventure surveys [44]. Cannabis related harms are assessed using a set of questions adapted from the Adolescent Cannabis Problems Questionnaire [50].

### Alcohol and cannabis knowledge

Alcohol related knowledge is assessed using a questionnaire adapted from the School Health and Alcohol Harm Reduction Project (SHAHRP)16 item Knowledge of Alcohol’ index [47]. The Cannabis knowledge questionnaire was adapted from the Cannabis Quiz and included 16 items [51].

### Intention to use alcohol and other drugs in the future

Five questions are used to assess student’s intention to use alcohol and other drugs in the ‘future’. Each question will require students to rate their intention on a five point likert scale labelled ‘very likely’, ‘likely’, ‘unsure’, ‘unlikely’ and ‘very unlikely’ [34].

### Mental health symptoms

Depression and Anxiety symptoms are measured using the Depression and Anxiety subscales from Brief Symptom Inventory (BSI), a standardised self-report symptom inventory. 7 items are designed to serve as a screen for depression, and 5 items for anxiety [52]. Participants are asked to rate the level of severity of each symptom in the last 6 month on a scale ranging from 1 ‘not at all’ to 5 ‘often’.

### Program evaluation

Upon completion of the *Climate Schools* program, students and teachers are asked to evaluate the program. Students are asked to indicate how acceptable, appropriate and enjoyable they found the program and to indicate how likely it is they will use the information taught in their own lives. Teachers are asked to give an overall rating of the program, rate it in comparison to other drug education programs, rate the educational quality of the program and to indicate how easy it was to implement, how well it held students attention, and how likely it was that they would use the program in the future.

For *Preventure*, students are asked to give an overall rating of the programs, and to indicate how much they liked the stories, how relevant they found the scenarios and how helpful they found the program, if they would recommend the program to others and to write down one thing they liked and didn’t like about the program.

### Implementation and treatment fidelity

All teachers delivering the *Climate Schools* program are asked to complete a logbook indicating which lessons and activities they completed with their class and to write down any adaption they made to the program. To ensure completion of the online part of the program, students are required to view the lessons in full and in order.

To measure treatment fidelity of the *Preventure* program, facilitators are evaluated by an Independent rater on 20% of sessions. The Facilitation Criteria Scale developed by Dr Conrod is used to assess treatment fidelity and has been employed in previous trials of Preventure [36]. Facilitators are evaluated on their adherence to the 14 core components of the Preventure Program (e.g., introduction to the program, goal setting, and decision-balancing exercise) on a 7-point Likert Scale ranging from “Poor” to “Excellent”. Facilitators are also evaluated on five core counselling skills deemed essential for the successful delivery of the interventions (listening, enabling, involving the entire group, being inquisitive and empathic). The co-facilitator is required to complete notes for each session to indicate how well the therapist adhered to the core components of the program, and to rate the overall engagement and concentration of the students. The facilitator is also required to complete a short therapist evaluation for each session.

### Statistical analysis

Baseline equivalence and attrition between groups will be examined using single-level analyses; one-way analyses of variance to examine normally distributed data, Chi-square to examine binominal data, and Mann–Whitney U-test to examine non-normally distributed data. To examine intervention by time interaction effects, mixed effects regression will be used due to the multi-level and hierarchical nature of the data. To account for intracluster correlations between schools, intervention effects will primarily be examined using hierarchical linear modelling (HLM) for normally distributed data and hierarchical generalized linear modelling using Poisson sampling for count data. Outcome variables will be centred at post-test to allow for comparisons between groups immediately after the intervention and growth terms will be analysed to determine the magnitude of the follow-up effects. Analyses will be conducted using the program HLM 6 [53]. If unconditional models reveal that less than 10% of systematic variance exists at the between-school level for any outcome variable, HLM will be abandoned and single-level analyses will be used [54]. For these variables, ANCOVAs utilising the SPSS GLM procedure will be conducted to account for any baseline differences that might exist between groups. Bonferroni adjustments will be made for multiple comparisons. Odds ratios, effect sizes and 95% confidence intervals will also be calculated.

## Discussion

The present study protocol presents the design of a randomized controlled trial evaluating the effectiveness of the CAP study; an integrated universal and selective approach to prevent alcohol misuse and related harms among adolescents. The primary aims of the study are to evaluate the efficacy of the CAP intervention in comparison to stand-alone ‘universal’ prevention and treatment as usual (TAU), in reducing the uptake and harmful use of alcohol, and reducing alcohol related harms. In addition, for high-risk students only, we aim to evaluate the efficacy of the selective personality-targeted *Preventure* program in comparison to stand-alone ‘universal’ prevention and TAU, in reducing the uptake and harmful use of alcohol, and reducing alcohol related harms.

### Strengths and limitations

The greatest strength of the CAP study is that it is the first study to evaluate the combined effect of two programs which have already proven their efficacy in preventing and reducing alcohol and other drug use. Secondly, the CAP intervention has the potential to maximize outcomes for both high- and low-risk youth and offers a way of preventing substance use at a whole population level. Finally, it overcomes traditional implementation obstacles to school-based prevention by being in part facilitated by the internet and in part manualised thereby minimising potential adaptation and enhancing implementation fidelity. In Australia there is currently no evidence of effective selective prevention and internationally there is no evidence of a combined universal and selective approach to substance use prevention.

One limitation of the study is that the information obtained from students including information on their risky behaviour is done so using self-reports. While this method might lead to measurements errors, self-report is the most favoured method of assessment for young people and has been found to have excellent discriminant [55] and predictive [56] validity with regards to substance-related symptoms [37,41]. There are currently no viable alternatives for data collection on alcohol use in an adolescent sample, as biological measures would not be appropriate in a sample at the early stages of alcohol use initiation [57]. Recommended methods to maximize the accuracy of participants self-reports are followed, e.g., visual prompts to assess quantity of alcohol consumption, and research staff independent of the schools carrying out paper and pencil assessments. In addition, participant anonymity and confidentiality is guaranteed, and it is emphasised that schools and parents would not have access to individual student data.

A second limitation regards the power of the trial. While this study is not powered to detect ES differences of below 0.3 between the integrated CAP intervention and the individual *Climate Schools* and *Preventure* programs, it maybe that only moderate effect sizes would justify the added burden of implementing both programs in schools. As an initial investigation, will seek to establish a moderate level of incremental validity of the integrated program over and above the significant effects of the two individual evidence-based programs. Future research may wish to recruit greater number of schools to detect smaller differences between the groups.

A final issue regards the selection of participants and stigmatization involved with screening participants to the selective *Preventure* intervention. To avoid stigmatization, neither the parents, teachers nor students at the intervention schools are explicitly informed about the selection process of determining which students are high-risk. This is an ethical issue which should also be taken into account if the program is implemented at other schools in the Australia in the future.

### Conclusion

Alcohol misuse by young people is a serious concern, yet few prevention strategies exist which are effective in decreasing alcohol use and related harms, and there are no programs which integrate universal and selective approaches to maximize the prevention outcomes at the population level. This article has described the study protocol, design and current implementation of a cluster randomized controlled trial to evaluate the effectiveness of the CAP study, a comprehensive and integrated universal and selective approach to prevent alcohol use and related harms among adolescents. The CAP intervention represents a utility that is practical, acceptable, fits within the school health curriculum, and is scalable to meet the needs of all schools in Australia.

Evaluation of the program will provide insight into the efficacy of the CAP intervention in Australian adolescents when compared to a stand-alone universal program and drug education as usual. If the program can reduce the target risk factors by levels equal or greater than that of the stand-alone *Climate Schools* program, then it will be a most significant contribution to promoting and maintaining the good health of the community in Australia and reducing the burden of disease, social costs, and disability associated with alcohol misuse and related harms.

## Competing interests

PC is one of the developers of the *Preventure* program. MT and NN are two of the developers on the *Climate Schools* program in Australia. Both programs are distributed not for profit. The other authors declare that they have no competing interests.

## Authors’ contributions

MT, PC, NN and TS are the Chief Investigators on the CAP study NHMRC grant in Australia. EB, NN and MT are responsible for ethics and clinical trial submission, recruitment of schools, and data collection. All authors will be involved in data analysis and reporting of the study results. All authors read and approved the final manuscript.

## Pre-publication history

The pre-publication history for this paper can be accessed here:

http://www.biomedcentral.com/1471-244X/12/118/prepub
